# Are We Ready for Cell Therapy to Treat Stroke?

**DOI:** 10.3389/fcell.2021.621645

**Published:** 2021-06-23

**Authors:** Fernando José Rascón-Ramírez, Noelia Esteban-García, Juan Antonio Barcia, Albert Trondin, Cristina Nombela, Leyre Sánchez-Sánchez-Rojas

**Affiliations:** ^1^Department of Neurosurgery, Hospital Cl nico San Carlos, Madrid, Spain; ^2^Regenerative Medicine and Advanced Therapies Laboratory, Instituto de Investigación Sanitaria San Carlos (IdISSC), Hospital Cl nico San Carlos, Madrid, Spain; ^3^Department of Surgery, Universidad Complutense de Madrid, Madrid, Spain; ^4^Department of Biological and Health Psychology, Universidad Autónoma de Madrid, Madrid, Spain

**Keywords:** stroke, diagnosis, therapy, cell therapeutic potential, clinical trial, administration route

## Abstract

Clinical trials of cell therapies that target stroke started at the beginning of this century and they have experienced a significant boost in recent years as a result of promising data from basic research studies. The increase in the information available has paved the way to carry out more innovative and varied human studies. Efforts have focused on the search for a safe and effective treatment to stimulate neuro-regeneration in the brain and to reduce the sequelae of stroke in patients. Therefore, this review aims to evaluate the clinical trials using cell therapy to treat stroke published to date and assess their limitations. From 2000 to date, most of the published clinical trials have focused on phases I or II, and the vast majority of them demonstrate that stem cells are essentially safe to use when administered by different routes, with transient and mild adverse events that do not generally have severe consequences for health. In general, there is considerable variation in the trials in terms of statistical design, sample size, the cells used, the routes of administration, and the functional assessments (both at baseline and follow-up), making it difficult to compare the studies. From this general description, possibly the experimental protocol is the main element to improve in future studies. Establishing an adequate experimental and statistical design will be essential to obtain favorable and reliable results when conducting phase III clinical trials. Thus, it is necessary to standardize the criteria used in these clinical trials in order to aid comparison. Shortly, cell therapy will be a key approach in the treatment of stroke if adequate and comprehensive levels of recovery are to be achieved.

## Introduction

Stroke is a condition in which oxygen, glucose, and nutrients flow is restricted or reduced in certain areas of the brain. The cellular response in the infarcted region is an inevitable consequence of cerebral ischemia. Regulated pathways are activated in these lesion areas that trigger tightly structured signaling cascades, but an unregulated pathway is also followed, known as accidental cell death, where this process is biologically uncontrolled. As a result, neurons in the ischemic penumbra become dysfunctional and undergo apoptosis. While it may be possible to offset the energy deficit in this region, opening the door to potential recovery from the insult, the adverse microenvironment, fluid accumulation, and damage to the extracellular matrix (ECM) complicates tissue preservation and ultimately, its restoration (Roll and Faissner, 2014). Different studies have shown that microglia promote spontaneous neurogenesis in Stroke by guiding neuroblasts to the site of injury (Lindvall and Kokaia, 2015). In turn, after the reduction in blood flow both acute and chronic vascular remodeling occurs. This vascular repair works in conjunction with neurogenesis to promote some recovery in the injured area (Zhao et al., 2008). Unfortunately, the spontaneous stimulation of neurogenesis and angiogenesis is insufficient to achieve complete repair, possibly due to the inflammatory environment, the problems in establishing functional connections and/or the lack of the necessary trophic support (Bond et al., 2015).

The epidemiology of stroke is constantly evolving and as a result, the implementation of new therapies in the acute phase can reduce mortality, although the incidence, prevalence, and morbidity remain high, making stroke a chronic disabling disease (Carmichael, 2016). Recent studies showed that 80% of patients manage to regain some but not all of the lost neurological functions and indeed, around 25–50% of patients become dependent for at least one activity of daily living 6 months after a stroke (Carmichael, 2016). Therefore, a priority of basic and clinical research into this pathology is the identification of therapeutic alternatives to enhance patient recovery. This has led to the current interest in cell therapy, a key strategy to enhance and complement endogenous restorative mechanisms in Stroke and to repair damaged tissue and restore neurological function (Gómez-Pinedo et al., 2018).

Cell therapy can be divided into two main processes, the replacement of pathological affected cells and the activation of endogenous mechanisms inducing tissue self-renewal (Singh and Rameshwar, 2018; Esteban-Garcia et al., 2020). Stem cells (SCs) have received significant attention because of their remarkable versatility. The main characteristics that define them are the capacity for self-renewal and transformation of their phenotype, or their progenies, both into differentiated or undifferentiated cells (symmetric and asymmetric cell divisions). The classification of SCs is based on their origin: adult stem cells (ASCs) and embryonic stem cells (ESCs).

ESCs are best-studied pluripotent SCs, and they can be isolated from the inner cell mass of blastocysts. On the other hand, ASCs mainly derives from the existing reserves in the adult tissues, or the cells present in the umbilical cord. One of the clearest and best-studied examples of ASCs are the cells that reside in the bone marrow (BM) that include (i) hematopoietic stem cells (BM-HSC) which have the capacity to give rise to all cells of the hematopoietic system, (ii) mesenchymal stem cells (BM-MSC) which can differentiate into bone, chondrocytes and adipose cells and (iii) endothelial SCs (Wislet-Gendebien et al., 2012). These cell groups are the most used in cell therapy due to their easy identification and expansion in culture, in addition to their therapeutic potential (immunomodulation, trophic, among others). Furthermore, a heterogeneous set of cells called, mononuclear cells (MNC) has been found. This subset is used to name all cells whose nuclei are not isolated or rounded and lack granules in the cytoplasm. This cell type can be extracted from the BM or peripheral blood (PBMC) and could contain different proportions of hematopoietic and mesenchymal stem cells. In most of the studies that will be discussed in this review, this has been used cell fraction, probably due to its easy isolation and the promising therapeutic functions demonstrated in different studies (Kucia et al., 2005; Li et al., 2006; Adler et al., 2011; Hatakeyama et al., 2020).

In this context, a current need is to improve the tools to predict accurately the adverse reactions of the treatments. Accordingly, the manipulation of adult human cells to generate induced pluripotential SCs (iPS cells) is a currently used technique, as these have been shown to have the same potential as endogenous cells, with the advantage of surviving in culture during prolonged periods (Anson et al., 2011; Buzhor et al., 2014; Hao et al., 2014).

In the past decade, SCs or progenitor cells have been studied in basic research due to their interesting effects as anti-inflammatory and anti-apoptotic agents, and as cells that enhance the presence of trophic factors or that stimulate neurogenesis and angiogenesis (Eckert et al., 2013; Wang et al., 2018). The promising results obtained prompted the initiation of clinical trials and led to the laying down of guidelines to design adapted preclinical studies (Boltze et al., 2019). However, despite the strong preclinical evidence available, the leap to clinical trials is complex. Most of the publications to date have served as ‘proof of concept’ or they have reported phase I or IIa trials. These are smaller studies mainly designed to assess the safety of the treatment (see Table 1). Some of them also included some evaluation of efficacy through functionality scales, such as the NIHSS (National Institute of Health Stroke Scale) to evaluate neurological deficits, as well as the modified Rankin Scale (mRS) and the Barthel Index (BI) that assess disability and dependence on activities of daily living, respectively. All of these are internationally recognized scales with significant clinical relevance (Chen et al., 2014; Zhang et al., 2019). Although most clinical trials have not followed ideal design parameters (multicentre, randomized, triple-blind, and controlled), they have provided useful safety data and they have allowed researchers to identify limitations that could not otherwise be anticipated. Therefore, this review will provide a critical insight into the clinical trials carried out using cell therapy as a treatment for Stroke, highlighting their limitations and benefits. Since the route of administration is a key parameter in administering therapy, the review is structured around this parameter.

**TABLE 1:** Summary of published articles on Stem Cell Therapy in Stroke.

**#**	**Report/year**	**Route**	**Study design**	**Stem cell type**	**Dose**	**Timing**	**n**	**Phase**	**Stroke type**	**F-U**	**1° outcome indicator**	**Cells origin**
1	Bang et al., 2005	IV	Randomized controlled	BMSCs	5 × 10^7^	4 weeks	30	I/II	Subacute	12	mRS, BI	PIC
2	Lee et al., 2010	IV	Open-label, observer blinded, randomized	BMSCs	5 × 10^7^	1–4 weeks	16		Acute	60	mRS, BI	PIC
3	Savitz et al., 2011	IV	Prospective open label	BMNCs	10 × 10^6^	1–3 days	10	I	Acute/Subacute	12	BI, NIHSS, mRS.	PIC
4	Honmou et al., 2011	IV	N/A	ExoMSCs	0.6–1.6 × 10^8^	36–133 days	12	I/II	Chronic	12	mRS, NIHSS,	PIC
5	Bhasin et al., 2011	IV	Nonrandomized	BMSC	50–60 × 10^6^	3–12 months	12	I	Chronic	6	NIHSS, FM, mRS	PIC
6	Bhasin et al., 2012	IV	Non-randomized	BM-MNCs	50–60 × 10^6^	3–24 months	12	I	Chronic	6	FMA, BI	PIC
7	Bhasin et al., 2013	IV	Non-randomized, unblinded, case control	BM-MSCs	50–60 × 10^6^	3–24 months	20	I/II	Chronic	24	MRI, BI, FM,	PIC
8	Díez-Tejedor et al., 2014	IV	Prospective, randomized, double-blind, placebo	Allogenic MSCs from adipose tissue	1 × 10^6^	2 weeks	20	IIa	Acute	24	mRS, NIHSS, MRI	Adipose tissue.
9	Prasad et al., 2014	IV	Multicentric, parallel group	BMNCs	280.75 × 10^6^	7–30 days	60	II	Subacute	6	NIHSS, BI, mRS.	PIC
			controlled									
10	Taguchi et al., 2015	IV	Non-randomized, open label	BMNC	2.5–3.4 × 10^8^	7–10 days	12	I	Acute	6	NIHSS, mRS, BI	PIC
11	Bhasin et al., 2016	IV	Randomized, controlled	BMNC+MSCs	50–60 × 10^6^	3–24 months	20	I	Chronic	6	FMA, BI	PIC
12	Hess et al., 2017	IV	Randomized, double-blind, placebo-controlled Multicentric	Multipotent adult progenitor cells	400–1,200 × 10^6^	24–48 h	65	II	Acute	12	BI, mRS, NIHSS	Multipot ent adult progenito r cells
13	Laskowitz et al., 2018	IV	Open label	Umbilical cord	3.34 × 10^6^	3–9 days	10	I	Acute	12	mRS	Allogeni c umbilical cord
14	Vahidy et al., 2019	IV	Single arm	BMNCs	Maximum dose: 10 × 10^6^ + escalated doses: 8–9 × 10^6^ + 5–8 × 10^6^.	1–3 days	25	I	Acute	24	mRS, NIHSS	Autologo us bone marrow
15	Tsang et al., 2017	IV	Randomized, controlled, double-blind	BMSC	4.57 × 10^7^	N/A	9	I/II	Chronic	14	GOSE BI	PIC
16	Levy et al., 2019	IV	Preliminary safety and efficacy	BMSCs	0.5–1.5 × 10^6^	Mean 6 months	36	I/II	Chronic	12	BI, ECG, CT.	Allogeni c MSCs.
16	Lv et al., 2013	IV. + IT	N/A	Umbilical-MS Cs	100 × 10^6^	7–30 days	60	I/II	Chronic	3–12	FMA	Umbilica l cord
17	Battistella et al., 2011	IA	Approximation study	BM-MNCs	1–5 × 10^8^	1–180 days	6	I	Chronic	6	NIHSS, Doppler, MRI, mRS, BI	PIC
18	Friedrich et al., 2012	IA	Non-randomized, non-controlled	BM-MNCs	15 ml	Mean 3–9 days	20	I	Subacute	6	NIHSS, mRS, CT,	PIC
19	Moniche et al., 2012	IA	Single blind controlled	BM-MNCs	159 m. (3.3 × 10^6^ CD34+)	Mean 5–9 days	10	I/II	Subacute	6	NIHSS, BI, mRS	PIC
20	Banerjee et al., 2014	IA	Prospective, non-randomized, open label	CD34+ stem/Progenitor	100 × 10^6^	7 days	5	I	Acute	6	MRI, NIHSS, mRS	PIC
21	Savitz et al., 2019	IA	Randomized, prospective, controlled, multicentre, blinded	BM/ALD-401		1–90 days	30		Acute	24	mRS, BI, RMN, NIHSS	PIC
22	Suárez-Monteagudo et al., 2009	IC-S	N/A	BMSC	N/A	1–10 years	5		Chronic	12	NIHSS, MRI, SF-36	PIC
23	Kondziolka et al., 2000	IC-S	Open label, observer blinded	Neurons	6 × 10^6^	6–72 months	18	I	Chronic	18	ESS, PET, BI SF-36, NIHSS	Cultured neurons
24	Kondziolka et al., 2005	IC-S	Open-label, observer blinded	Neurons	5–10 × 10^6^	>6 months	14	II	Chronic	12	ESS, PET, BI SF-36, NIHSS	Cultured neurons
25	Chen et al., 2014	IC-S	Randomized single-blind	PBSCs	3–8 × 10^6^	6.60 months	15	I	Chronic	12	NIHSS, mRS	PBSCs
26	Kalladka et al., 2016	IC-S	Open-label, single site	CTX-DP	2–20 × 10^6^	6–60 months	13	I	Chronic		NIHSS, mRS, MRI, BI, Ashworth	Immortalized h. stem-cell
27	Zhang et al., 2019	IC-S	Cohort	NSI-566 1°adherent neural stem cells	A = 1.2 × 10^7^ B = 2.4 × 10^7^ C = 7.2 × 10^7^	Mean 494 days	9	I	Chronic	24	CT, fMRI, PET, DTU	Cells derived from human fetal spinal cord
28	Steinberg et al., 2019	IC-S	Open label single arm	BMSC SB623	2.5–10 × 10^6^	6–60 months	18	I/IIa	Chronic	24	NIHSS, mRS, ESS, FM	Allogeni c bone marrow derived.
29	Feng et al., 2014	IT	N/A	UC-MSCs	100 × 10^6^	14–30 days	50		Subacute	3	NIHSS, FMA	Lianonin g blood center

## The Selection of the Route of Administration

When considering the design of the clinical trials, one of the main questions to define is the route of administration. This is usually related to the pathological stage of the Stroke to which the treatment is directed, as well as to other aspects like the level of safety of the treatment, the type of cells, or the dose. The parenteral route of administration includes different modalities: intra-arterial (IA), intravenous (IV), intraperitoneal (IP), or intranasal (IN). These routes have the advantage of being less invasive, safer, and more comfortable for the patient than the local or intracerebral (IC) route of administration (Hao et al., 2014). Another relevant issue to take into account is the possible adverse effects (AEs) of these innovative treatments. Among them, we must differentiate among effects derived from: (i) the treatment, (ii) the procedure, or (iii) the route of administration (Cui et al., 2015; Boltze et al., 2019). For this reason, we will structure the different published clinical trials according to their route of administration, since each route has certain implications and therefore, those in which the same route is used will contain similarities.

### Intravenous (IV)

The IV route of administration is that often most used in clinical studies of cell therapy for ischemic stroke. This route involves infusion through a central or peripheral venous catheter, similar to blood transfusion, and its ease of administration allows it to be used when administering treatments at very early stages after stroke. Most of the clinical trials that used IV administration did not inject cells in a single dose but rather, in regimes involving 2 to 5 injections. The minimal invasiveness of this route permits repeating administrations with little risk to the patient. However, there are possible complications, such as pulmonary embolisms or thrombi due to the accumulation of infused cells (Boltze et al., 2015). To date, 14 studies have been published that have used IV administration.

One of the pioneering clinical trials using cell therapy in Stroke was that carried out in 2005 (Bang et al., 2005). The study included 5 experimental and 25 control subjects, from whom BM mononuclear cells (BM-MNCs) were extracted at the sub-acute stage that were expanded in culture to obtain BM-MSCs. The treatment consisted of injecting 5 × 10^7^ MSCs at two time-points, with efficacy measured with the mRS and BI functional scales, and by magnetic resonance imaging (MRI), while safety was measured by the absence of allergic reactions or local/systemic complications. However, there was high experimental mortality after a one-year follow-up, with just 5 experimental subjects and 5 controls undergoing the MRI scan. The follow-up data indicated a reduction in neurological deficits and an increase of the functional recovery indices. Their most prominent finding was a non-significant decrease in infarct size and ventricular dilation in the MSC group relative to the control group (Bang et al., 2005). Although this trial was not a blind trial, it was a randomized and controlled trial, and given its pioneering status, it is considered a milestone in the field. There has been no other clinical trial to treat stroke using the same cell type, except for a study comparing the use of MNCs and MSCs (Bhasin et al., 2013).

In another trial, 36 control subjects and 16 randomized experimental patients received two doses of 5 × 10^7^ MSCs each, between weeks 5 and 7 after the stroke, and they were followed for up to 5 years (Lee et al., 2010). This was one of the longest studies in terms of duration, and it demonstrated long-term benefits of MSC therapy in terms of functional outcome and survival, although they suggested shortening the administration time and bringing it much closer to the ischemic event (Lee et al., 2010).

One author who has devoted his career to this field is Dr. A. Bashin. In an initial study, 12 chronic stroke patients received IV injections of expanded BM-MSCs who were followed for 24 months. The aim was to identify possible long-term AEs, which were ultimately not reported, and the results showed a trend toward an improvement in the experimental group relative to the controls (Bhasin et al., 2011). One year later, the same research group performed a similar clinical trial using CD34^+^ cells extracted from BM and isolated by cytometry. This process took only 2 h, allowing the cells to be collected and administered on the same day. The strong variability in the volumes extracted from each patient and the number of CD34^+^ cells obtained was quite notable, and on average, of 52–58 × 10^6^ cells obtained only 0.3% were CD34^+^ cells. A dose of 54.6 million cells was administered in a single 250 ml infusion, thereby minimizing the main risk of this route of administration that is the formation of thrombi. Subtle changes in the cortical reorganization were observed when assessed by functional imaging techniques: BOLD sequences, dependent on the level of blood oxygenation; and DTI, diffusion tensor images. Although these measures did not provide decisive results, they were considered good complementary alternatives to other scales to quantify efficacy that might provide more solid data (Bhasin et al., 2012).

Using a similar cell procedure in a study carried out almost simultaneously, a controlled clinical trial administered one of the highest doses of cells ever published: 10 million cells/kg weight of CD34^+^ cells (Savitz et al., 2011). Since patients were treated in an acute phase, the cells were extracted from the BM aspirate and isolated by cytometry in a process that lasted 2 h. The study recruited patients diagnosed with acute or subacute stroke (between 0 and 60 h post-stroke) and after 6 months, there was a trend toward improvement in the experimental patients in the absence of AEs (as assessed through blood, liver, and renal parameters: (Savitz et al., 2011). Significantly, 10 patients were included out of the 786 who attended the service in the trial period, which reflects the difficulties in recruiting patients who meet all the inclusion criteria, as observed in other trials.

In a third clinical trial, Bashin compared the effect of MSCs and MNCs with a control group in 2013 (*n* = 20). The study followed their previous protocol on a larger sample of 20 experimental patients distributed into two groups, administering an infusion with 50–60 million cells diluted in 250 ml: (a) 14 patients receiving MNCs (isolated from a BM aspirate and selected by flow cytometry), and (b) 6 patients receiving MSCs (firstly isolated by cytometry and then expanded in culture for approximately 23 days). The authors explain: “*The mean CD34^+^ count of MNC was 0.28% with mean 55.4* × *10 to the 6th millions cells whereas mesenchymal cells expressed CD90, CD73, CD105 and were negative for class HLA II. The mean CD90, CD73 and CD105 were 61%, 57.1% and 40% respectively.*” The MNC group showed more activity in the primary premotor and parietal areas relative to the controls, although the differences between the groups did not reach thresholds of significance (Bhasin et al., 2013). From all these trials, we conclude it is not possible to detect differences in the effectiveness of MSCs or MNC CD34^+^ cells. Thus, it is necessary to continue investigating the differences in the effect of administering heterogeneous populations like CD34^+^ cells or homogeneous populations like MSCs.

Two multicentric studies have been reported (Prasad et al., 2014; Hess et al., 2017). One of them was published in 2014, from the 120 randomized sub-acute stroke subjects, 117 completed the one-year follow-up. The experimental group was administered autologous BM-MNCs (with a mean of 280.75 million mononuclear cells infused, containing 2.9 million CD34^+^ cells), and efficacy was determined using the BI and NIHSS questionnaires. The results did not show differences between the groups at either a 90- or 180-day follow-up, either in functional tests or in neuroimaging scans. Safety parameters were verified using PET and electroencephalography, with no signs of toxicity, neoformations compatible with tumor masses, or the activation of the cell cycle (Prasad et al., 2014). Another multicenter study in 2017, published the results of 129 randomized patients, half of them received multipotent adult progenitor cells, and the other half received placebo. They showed that the use of these cells was safe and well-tolerated, but they also saw that there were more excellent results (measured by different scales) with significant differences in favor of those patients who received cell therapy (Hess et al., 2017).

In parallel to previous studies, a phase I/IIa clinical trial was performed to evaluate the efficacy and safety of autologous BM-MNCs IV transplantation (Taguchi et al., 2015). The work included 12 non-randomized patients organized into a low dose and a high dose group. Both initially and 7 days after admission, their assessment included the NIHSS questionnaire, while the 30-, 90- and 120-day assessments used the mRS scale and the BI, together with neuroimaging scans. No differences were evident between the groups for any of the scales, nor were any changes in the levels of CD34^+^ cells in the peripheral blood. However, two patients registered AEs: pneumonia and recurrent cerebral infarction (Taguchi et al., 2015). This is the second study to include a dual experimental group, in this case with two different doses. However, the non-randomized and non-blind design, a limited sample size, and the evidence of two relatively frequent AEs through this route of administration limited the scope of its contribution.

In 2016, Bashin’s group published a fourth clinical trial using BM-MNCs tested in an experimental (*n* = 10) and a control group (*n* = 10). For the first time, apart from the usual neurological scales, the protocol included an assessment of paracrine signaling as an approximation for treatment efficacy. The authors determined the levels of vascular endothelial growth factor (VEGF) and brain-derived neurotrophic factor (BDNF), although their relationship with the number of CD34^+^ cells was not determined. Even though the results did not demonstrate efficacy, they witnessed some paracrine activity of BM-MNCs (Bhasin et al., 2016).

A different set of clinical trials were notable for using adipose tissue-derived MSCs (ADSCs: (Díez-Tejedor et al., 2014), multipotent adult progenitor cells (Hess et al., 2017), and umbilical cord-derived cells (UC-MSCs; Tsang et al., 2017; Laskowitz et al., 2018). These trials employed IV administration of cells taken from less conventional sources and applied the cell therapy at the acute phase of Stroke. Specifically, the trial using ADSCs is one of the few studies to propose a single application (1 million cells/kg weight administered at 4–6 ml/min) two weeks after stroke (Díez-Tejedor et al., 2014). In this Phase IIa, pilot, single-center, prospective and double-blind study, 20 patients were randomly assigned to the control and experimental group. The main variable was safety, measured according to the appearance of AEs and the generation of tumors. However, the results of the primary safety variables and the secondary efficacy variables were not published (Díez-Tejedor et al., 2014).

Levy et al. published the use allogeneic and not autologous, which is the most common due to the safety issues (an autologous transplants will be generally considered be safer than allogeneic ones). Their simultaneous dual-phase I and II study on chronic stroke patients used MSCs in a dose-escalation design and established a one-year follow-up period. The results demonstrated safety and suggested functional improvement in the experimental group, although several patients were excluded. The study design did not include a control group (Levy et al., 2019).

The trial with the largest number of participating centers to date was published in 2017. It was a coordinated multicenter trial conducted in 33 centers in the United Kingdom and the United States, using multipotent adult progenitor cells. This was a careful and accurate, controlled, randomized, double-blind study, and with a stratified assignment of the experimental group: a single dose of 400 million or 1200 million cells administered 24–48 h after the onset of symptoms. Although there was no significant evidence of efficacy, the safety of the treatment was robustly assessed, and AEs of special importance were not reported despite the high doses administered (Hess et al., 2017).

Similarly, UC-MSCs were used in an aleatory, controlled, double-blind phase I/II trial on 9 chronic patients (Tsang et al., 2017). Treatment consisted of two-doses of 4.57 × 10^7^ MSCs given 6 and 10 weeks after recruitment. The study analyzed various markers of MSCs to characterize the treatment while assessing neurological function and functional independence with the BI and Glasgow Outcome Scale for up to 60 weeks after injection. The efficacy of the treatment was determined based on the non-significant improvement in the experimental group versus the controls, providing useful criteria for the future design of phase III trials (Tsang et al., 2017).

The latest publication in this field was the first trial to address effect size (Vahidy et al., 2019). A Phase I study was carried out on 30 patients with acute stroke infusing the treatment 24 to 72 h after the appearance of symptoms. This may be fundamental to determine a clinical effect, as proposed previously (Lee et al., 2010). The volume of BM harvested was 2 ml/kg to infuse a maximum MNC dose of 10 million cells/kg, although 4 patients received 8 to 9 million cells/kg and another 3 patients received between 5 and 8 million cells. Various AEs were recorded during the one-year follow-up period, which modified the exclusion criteria. Three patients suffered an expansion infarction and one had an episode of hypotension (Vahidy et al., 2019).

Taken together, all these clinical trials indicate that the design of the trials is becoming more and more solid, without yet reaching an optimal scenario. Regarding the origin of cells, cell therapy is evolving toward other forms that offer the same benefit but are more accessible; the administration of exosomes derived from MSC is already being tested in animal models (Chen et al., 2016), serum from the culture of autologous MSCs (Honmou et al., 2011) or allogeneic UC blood (Laskowitz et al., 2018). The studies published to date in which IV administration has been used share the main variable: safety. This is a fundamental aspect that has been witnessed in the majority of studies, even when different cell types are used. Efficiency, on the other hand, still requires some verification. Various authors have tried to demonstrate efficacy not only with scales but also, by evaluating growth factors or effect size. This is probably the aspect that most urgently needs to be strengthened in order to define the parameters that need to be assessed in future clinical trials.

### Intra-Arterial (IA)

The IA route of administration involves cannulation of the ipsilateral middle cerebral artery through a puncture in the femoral or radial artery. This procedure is usually performed by an interventional radiologist, and it requires local anesthesia and patient monitoring throughout the entire process. There are five clinical trials in the literature that have used this route of administration, three of which focused on sub-acute stroke (3 to 9 days after the stroke) and two at a more advanced stage, 11–17 days and 90 days after the stroke. Four of the five studies infused autologous BM-MNCs within hours of extraction and one employed a population of selected BM-SCs. In general, this strategy has limited benefits as it is more invasive than IV and not as precise as IC administration (Cui et al., 2015).

The first pilot trial using this approach was published in 2011 and it was an approximation study without a control group or a blind design (Battistella et al., 2011). The doses ranged from 1 to 5 × 10^8^ cells infused 90 days after the stroke, yet the main limitation of the study was the sample size, involving only 6 patients. However, among the variables assessed were blood analysis and marked cell scintigraphy, which could be of interest when determining the effectiveness of the treatment.

The next trials conducted with IA administration followed quickly, the first including 8 experimental subjects and 9 non-randomized, blind, control subjects (Moniche et al., 2012). A total of 1.559 × 10^8^ cells were infused, of which 3.38 × 10^6^ were CD34^+^. The cells were administered 5 to 9 days after stroke (sub-acute) and the patients were followed for 6 months, evaluating them by NIHSS, BI, and mRS. In this study, 2 subjects suffered seizures 3 months after treatment, although there did appear to be a correlation between the biological parameters and the neurological scales. After the 6 month follow-up, markers of interest were identified that included: (i) matrix metalloproteinases 2 (MMP-2); (ii) granulocyte-macrophage colony-stimulating factor (GM-CSF); and (iii) platelet-derived growth factor-BB (PDGF-BB). It was concluded that the injection of BM-MNCs induced a decrease in the serum levels of these factors, which may be associated with better functionality, even 3 months after administration (Moniche et al., 2014). Although the results showed hardly any significant difference between the two groups, they did highlight certain parameters that were worth exploring. In another trial (Friedrich et al., 2012), a single experimental group of patients received an infusion of 15 ml of cells 3 to 7 days after stroke. The study mainly focused on safety and the results were assessed by computerized axial tomography (CAT) and magnetic resonance imaging (MRI), showing no significant anatomical or structural changes that might reflect an AE.

A prospective, non-randomized, open-label study was carried out 2 years later that was conceived as a proof of concept (Banerjee et al., 2014). The cell therapy involved autologous transplantation of previously immuno-selected CD34^+^ progenitors, applied in the range of 1.2 to 2.79 × 10^6^ cells. Although initially, the study started with a total cohort of 76 patients with severe acute ischemic stroke, only five reached the end of the 180-day follow-up. The effects of the treatment were measured by neuroimaging and using clinical scales but they did not provide efficacy results. However, no treatment-related AEs were recorded.

Finally, a recent trial deserves to be mentioned for its experimental design, which contemplated all the main aspects: randomized, prospective, controlled, multicentre, and blind (Hess et al., 2017; Savitz et al., 2019). The experimental group received the cells infused into the carotid artery 11 to 17 days after the stroke, whereas in the sham group this was simulated 13 to 19 days after the stroke. The main parameters, the safety of the route of administration and the cell type, were assessed by the lack of alterations or AEs derived from the treatment. By contrast, there were no differences in the effectiveness parameters. This study served to establish precedents about the parameters to use in the design of subsequent clinical trials.

Most of the studies presented that used this route of administration included neuroimaging techniques and clinical scales, in addition to other molecular parameters. Although none provided conclusive results on the efficacy of the treatment, the combination of evaluating molecular parameters together with neurological scales or neuroimaging could be key to gaining a better understanding of the efficacy of these treatments. In parallel, these studies once again highlight the need to establish a more standardized experimental design, eliminating intra-assay variability.

### Intracerebral (IC)

Most of the studies using IC treatments coincide in an open-label or single-blind design, as it is not possible to perform a placebo brain intervention to meet the double-blind criteria. Furthermore, just one of the 7 published studies included a control group, which makes it difficult to establish whether the effect of IC cell therapy is due to the treatment or not, as occurs in another clinical trial on cell therapy (Kondziolka et al., 2000, 2005; Suárez-Monteagudo et al., 2009; Chen et al., 2014; Kalladka et al., 2016; Steinberg et al., 2019; Zhang et al., 2019). The iconic clinical trial on cell therapy for stroke was not only the first of its kind, but it involved the administration of neurons differentiated *in vitro* from the human NT2/D1 cell line along with a conditioning medium (Kondziolka et al., 2000). The cells were administered at a dose of 2 million cells, divided into three implants (20 μl/implant) distributed over three trajectories. Functional assessments were based on the NIHSS, BI, European Stroke Scale (ESS), and neuroimaging scans (PET and MRI). One of the outstanding results of this study was the absence of complications during surgery, particularly given the risks associated with this invasive procedure. Also, there were no AEs after a 24 week follow-up period. In a later study, attempts were made to restore lost motor tissue after an ischemic event and a second trial was proposed using the same cell type (Kondziolka et al., 2004), an ambitious and pioneering study that administered cells differentiated into neurons.

Another open clinical trial was published a few years later (Suárez-Monteagudo et al., 2009) that had several peculiarities: (i) the sample included 5 patients with neurological sequelae caused 1 to 10 years previously; (ii) the volume of selected BM-MNCs administered ranged from 40 to 60 ml; (iii) the neurological evaluation involved the use of the NIHSS, BI, and Scandinavian Stroke Scale; and (iv) the impact of surgery was measured with the Mini-Mental State Examination Scale (MMSE) and other neuropsychological tests. This study presented some risks in terms of design, derived from the small sample size and from the selection of patients at different stages of stroke, which meant that the complications may be more heterogeneous and the results may not be conclusive. In any case, the study offered further support for the safety of this route of administration, as no AEs or complications in previously unexplored functions were evident.

A subsequent clinical trial was based on preclinical studies, and it recruited 30 patients that were distributed between the control and experimental groups (Chen et al., 2014). The latter received a dose of 15 μg/kg G-CSF, a factor that stimulates the mobilization of CD34^+^ from peripheral blood stem cells, and they were subsequently isolated by leukapheresis before cell implantation, which makes the study results difficult to interpret. Improvements in the NIHSS, ESS, and EMS (European Motor Subscale) were observed in the experimental group, as well as a reduction in the affected volume determined by tractography. This was the first time that the stimulating factor was administered as concomitant medication. To better understand the effect of this novelty it would have been desirable to count on blood analysis post-surgery.

One of the first studies with an allogeneic transplant (Kalladka et al., 2016), used a commercial line of immortalized human neural stem cells (CTX0E03) from ReNeuron. The study involved delivery by stereotactic ipsilateral putamen injection from 6 to 60 months after ischemic stroke and it was designed with escalating doses: single doses of 2 million, 5 million, 10 million, and 20 million cells. This open-label, single-site study included 13 patients that did not develop any AEs related to the administration procedure or the implanted cells, regardless of the dose. Besides, the neurological scales showed a trend toward improvement, particularly in arm spasticity, leg spasticity, activities of daily living, and NIHSS.

In a recent clinical trial using MSCs (Steinberg et al., 2019), of the 379 patients initially evaluated only 18 met the inclusion criteria: (1) at least 6 months post-ischemic stroke; and (2) chronic motor deficits/sequelae. Trials using local IC administration usually recruit chronic stroke patients, guaranteeing the stability of the patient and reducing the risks associated with the intervention in sub-acute or acute stroke patients. In this recent study, BM-MSCs were implanted and each of the three groups received a single dose of 2.5, 5.0 or 10 × 10^6^ cells, which were implanted into the peri-infarct sub-cortical stroke region guided by an MRI stereotactic technique. After a 24-month follow-up, 7 patients experienced 9 serious AEs (SAEs), with no apparent trend between cell dose and the frequency of SAEs. The AEs included pneumonia, urinary tract infection, sepsis, or subdural hematoma, which although they resolved without sequelae and were not related to the treatment or procedure, were considered serious. It could not be established whether the AEs were linked to the surgery or not, or if they were exclusively related to the patient’s clinical condition (or associated with concomitant pathologies).

The latest of the published trials (Zhang et al., 2019) treated patients with NSI-566 cells, a cell product from Seneca Biopharma that has been used previously for amyotrophic lateral sclerosis and spinal cord injury. This is a stable, primary adherent neural SC line derived from a single human fetal spinal cord, and they have been epigenetically expanded without genetic modification. The objective of this trial was to evaluate the viability and safety of these cells in the treatment of hemiparesis due to chronic motor stroke. The design consisted of three patient groups that each received injections of 1.2, 2.4, or 7.2 × 10^7^ cells, together with immunosuppressor therapy with tacrolimus for 28 days. Treatment was well tolerated at all doses and MRI studies showed evidence of cavity filling due to the formation of new neural tissue in all the patients.

In general, clinical trials based on the IC pathway require more differentiated and innovative cell types (as opposed to MNCs or CD34^+^ cells), and all the studies to date have been performed on patients with chronic stroke due to their clinical stability. Furthermore, most studies report more AEs or complications than IV or IA administration, since the intervention itself carries a higher risk. Unfortunately, the results of the clinical trials using an IC route of administration do not support a greater efficacy relative to the other two, although the treatment is delivered directly to the target area.

## Discussion

In this review, we have analyzed the design and results of some twenty studies and clinical trials into the use of SCs to treat stroke patients. Although they are very varied and heterogeneous, many of them emphasize the safety but not necessarily the efficacy of these therapies. In other words, many of the studies were carried out in phases I or II, and they have provided necessary and convincing information to advance the study of these treatments. This review highlights the variety of study designs, scales, and criteria used in the clinical trials published to date. Consequently, it becomes hard to determine the pros and cons of each approach. Future studies should aim to overcome this situation and consider the application of a common set of parameters, such as those contemplated below.

### Study Design

*Randomization*: even when dealing with small cohorts, group assignment must be randomized to avoid assignment bias and to comply with the principle of causality. Not all studies discussed above meet the randomization criteria.

*Blindness*: Investigators, clinicians, and patients (triple-blind) need to be naïve to the participant’s allocation. It is advisable to have a single member of the research team in charge of administering the treatment and not participating in inpatient assignments, or assessments at baseline or follow-up (Tsang et al., 2017). The advantage of the triple-blind design concerning the blind design is the limitation of observer bias as opposed to just limiting the placebo effect (single-blind design).

*Control group*: To comply with the principle of comparison it is essential to include a control group to assess the weight of the treatment in the result. Besides, this enables effects that occurred during the experiment but are not attributable to the treatment to be ruled out or explained, as well as helping to determine the feasibility of the trial. In short, establishing a control group in all the studies would allow us to get more solid efficacy results, as seen in some of the valid examples mentioned here (Bhasin et al., 2013, 2011, 2012).

*Multicentre*: Heterogeneity of the sample increases the external validity of the data, making it susceptible to extrapolation to further populations. To date, virtually most of the studies published have been conducted at a single-center, except three of them (Hess et al., 2017; Savitz et al., 2019).

*Sample size*: Most studies in this field involved small cohorts (*n* < 65), often due to recruitment difficulties. The wide variety of baseline clinical conditions and stroke locations reduces the homogeneity of the experimental cohorts. Future protocols should consider the mechanisms available to maximize population homogeneity.

Smaller sample sizes were more frequent in trials in which the IC route was used for administration (i.e.,: those that require brain surgery for cell administration). However, trials have shown that IC therapy is safe in most cases and no significant risk is associated with this procedure compared to the other options.

### Treatment Characteristics

*Doses*: The optimal dose and timing of administration of cell therapy have yet to be established. This may depend on different factors, such as the type of cell administered, the route of administration, the patient’s characteristics, and the stage of the pathology. Parenteral routes of administration (IA, IV) require more SCs to achieve their goal, which could augment some of the side-effects. However, preclinical research showed that one of the AEs related to these routes of administration (pulmonary embolism) is controlled by managing the cell number administered and the infusion rate (Cui et al., 2015). By contrast, local administration through the IC route requires fewer cells, since the treatment is introduced directly into the brain parenchyma. However, it is an invasive technique that might be associated with additional risks.

As we have seen throughout this review, the doses used in clinical trials vary widely, from 1 to 2 million cells/kg of body weight to 50 to 60 million cells/kg. Also, the optimal timing for transplantation depends on the changing microenvironment of brain tissue after stroke. Early implantation of cells after a stroke likely has a neuroprotective effect due to its ability to counteract toxicity and inflammation. Conversely, cell transplantation 2–4 weeks after a stroke could have consequences on endogenous neuronal repair, favoring plasticity, angiogenesis, and neurogenesis, which are more intense at that time (White et al., 2000; Park et al., 2006; Stroemer et al., 2009).

*Cell origin:* There is no consensus as to the most appropriate cell phenotype to treat a stroke. Although the vast majority of clinical trials have used BM-MNCs through different routes of administration, other types of cells provide similar results, including (i) neurons differentiated from a line of neural precursors; (ii) MSCs expanded in cell cultures; and (iii) selected CD34^+^ cells that confer an autologous nature to the treatment (Shyu, 2006). This leads to another discussion regarding the type of transplant: autologous versus allogeneic. The “safety” of autologous transplants, as opposed to allogeneic ones, is highly dependent on the cell source, since BM-MNCs from an HLA-non-compatible donor are not safe, whereas an MSC non-compatible donor does appear to be relatively safe. Despite the many advantages of autologous transplants, allogeneic transplants offer homogeneity of the treatment when the treatments are “manufactured” according to Good Manufacturing Practice (Banerjee et al., 2014).

*Cell survival:* Different factors could affect cell survival and that has appeared in different studies. The phase of the stroke at which we apply the treatment could limit cell survival due to restricted blood flow, oxygen deficiency, trophic factors, oxidative stress, or inflammation in the affected region (White et al., 2000). In an attempt to reduce these effects, some studies incorporated genetic modifications or growth factor overexpression to promote the MSC or NSC survival (Lee et al., 2007, 2009, 2010, 2015). One of the most recent applications is that of cell encapsulation in biomaterial scaffolds before implantation into the ischemic region (Jin et al., 2010; Sanchez-Rojas et al., 2019).

Reaching a clear conclusion about the route of administration and the optimal cell type to use is perhaps one of the difficult issues to resolve, with many arguments in different directions.

### Tests to Determine the Efficacy

Almost all the trials reviewed measured safety as one of the main parameters, with only a few studies showing the efficacy of the treatment. This difficulty in part emanates from the heterogeneity of the neurological sequelae in the patients, even though using validated scales will result in scores with a more “global” meaning (less sensitive to the changes). In other words, many studies do not report statistically significant differences but just “trends toward improvement.” Consequently, future studies should include scales with higher discriminability scores. Specifically, it might be useful to add more discriminatory neurological scales, such as those that independently assess functions like language, motor strength, gait and spasticity, range of joint movement, podometric values, or cognitive status. Such scales have already been contemplated in at least one study (Kalladka et al., 2016). Finally, anatomical neuroimaging techniques can evaluate the safety and they can be used in conjunction with the evaluation of efficacy. Combined anatomical and functional MRI sequences, such as the BOLD and DTI signals, offer a direct measure of the integrity of connections, activation patterns, and metabolism that may be related to the efficacy of the treatment in the mid-and long-term.

### Safety Considerations

Currently, safety is demonstrated in virtually all published clinical trials. Only sporadic adverse effects related to the procedure, not due to the treatment, have been recorded.

Adverse effects from the procedure range from mild to severe. All of them could be minimized according to the limitations of the route of administration used. At the same time, the analysis of adverse effects per patient is recommended to clarify the causality of said adverse effects and their possible relationship with concomitant diseases.

Moll et al. establish the importance of identifying coagulopathies in patients susceptible to receiving treatment with cell therapy and previously establishing thromboprophylaxis, thus avoiding harmful effects (Moll et al., 2019, 2020). Along the same lines, Caplan et al. analyze the effects that the activation of the immune response, both innate and adaptive, can have on the effect of stem cell treatment (Caplan et al., 2019), comparing both preclinical and clinical data.

Despite these significant advances, we must not forget that the clinical trials published to date are, in many cases, pilot studies in small samples. Large-scale, multicenter, large-sample clinical trials are currently underway, which will provide relevant clinical information on safety and related issues.

### Follow-Up Time

Despite the heterogeneity in the follow-up times employed in the different studies, it seems reasonable to propose a minimum follow-up of one year, despite the associated costs, since most data loss occurs in the 3–6 month follow-up period. Furthermore, in clinical trials, the safety aspect may require a 2 year (Steinberg et al., 2018) or 4-year follow-up (Bhasin et al., 2017) to obtain truly reliable data. Our research group is conducting a blind, controlled, randomized phase IIa clinical trial (CELICTUS) of transplantation with allogeneic fatty tissue-derived ADSCs in which we have tried to incorporate the findings of interest from this literature review, and from which we expect to publish promising results soon.

## Conclusion

Cell therapy as a treatment for stroke appears to be safe based on the data obtained in clinical trials. There is a trend toward clinical improvement in the sequelae of the patients studied, but without reaching consistent statistical significance. Future clinical trials should aim to incorporate unified criteria. We believe that the parameters indicated in this review may serve as a “basis to define future clinical trial protocols” to obtain more robust and reliable results. In this sense, it is essential to find a balance between the risks and benefits of this promising treatment for ischemia, which is why performing more research is required.

## Author Contributions

LS-S-R: study concept and design. FR-R and NE-G: acquisition of the data. FR-R, NE-G, LS-S-R, and AT: analysis and interpretation of the data. LS-S-R and CN: drafting of the manuscript. JB, CN, and AT: critical revision. CN: submission. All authors contributed to the article and approved the submitted version.

## Conflict of Interest

The authors declare that the research was conducted in the absence of any commercial or financial relationships that could be construed as a potential conflict of interest.
